# Revealing the Synaptic Hodology of Mammalian Neural Circuits With Multiscale Neurocartography

**DOI:** 10.3389/fninf.2019.00052

**Published:** 2019-07-30

**Authors:** Erik B. Bloss, David L. Hunt

**Affiliations:** Howard Hughes Medical Institute, Janelia Research Campus, Ashburn, VA, United States

**Keywords:** neurocartography, array tomography, synapse, hippocampus, electron microscopy, dendritic spine, synaptic clustering

## Abstract

The functional features of neural circuits are determined by a combination of properties that range in scale from projections systems across the whole brain to molecular interactions at the synapse. The burgeoning field of neurocartography seeks to map these relevant features of brain structure—spanning a volume ∼20 orders of magnitude—to determine how neural circuits perform computations supporting cognitive function and complex behavior. Recent technological breakthroughs in tissue sample preparation, high-throughput electron microscopy imaging, and automated image analyses have produced the first visualizations of all synaptic connections between neurons of invertebrate model systems. However, the sheer size of the central nervous system in mammals implies that reconstruction of the first full brain maps at synaptic scale may not be feasible for decades. In this review, we outline existing and emerging technologies for neurocartography that complement electron microscopy-based strategies and are beginning to derive some basic organizing principles of circuit hodology at the mesoscale, microscale, and nanoscale. Specifically, we discuss how a host of light microscopy techniques including array tomography have been utilized to determine both long-range and subcellular organizing principles of synaptic connectivity. In addition, we discuss how new techniques, such as two-photon serial tomography of the entire mouse brain, have become attractive approaches to dissect the potential connectivity of defined cell types. Ultimately, principles derived from these techniques promise to facilitate a conceptual understanding of how connectomes, and neurocartography in general, can be effectively utilized toward reaching a mechanistic understanding of circuit function.

## Introduction

The mammalian brain is an impressive computational device, integrating external sensory stimuli with various internal states to select and implement adaptive behaviors. Can we provide a mechanistic explanation for how the brain performs each aspect of these computations? Just as the interactions of an atom can be understood by the orbital structure of its valence electrons, or the activity of an enzyme by the amino acid structures of its catalytic domain, one approach toward understanding the functions of the brain is to study the structural organization of its constituent circuits, cell types, and synapses. Only when we fully understand synaptic hodology and the dynamic interactions between circuit elements can we derive a mechanistic understanding of neural circuit computations (Denk et al., 2012).

**FIGURE 1:**
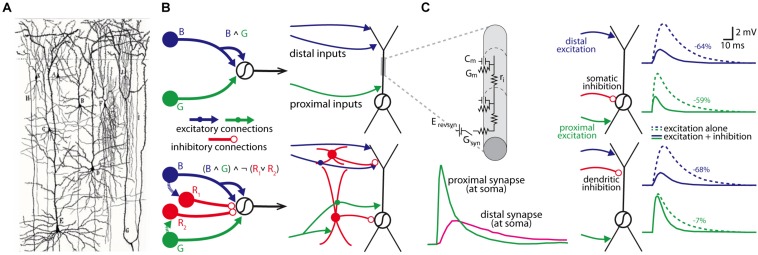
Individual neurons are organized into circuits that perform computations. **(A)** Cajal’s neuron doctrine was inspired by hand-drawn reconstructions of individual neurons stained by Golgi’s staining methodology (drawing is from Cajal’s work on human neocortex; Taken from DeFelipe (2017); panel kindly provided by Dr. Javier DeFelipe). **(B)** Two McCulloch-Pitts style logical circuits performing an “and” computation (B ∧ G; top) or a type of “not” computation ((B ∧ G) ∧ ^¬^ (R_1_ ∨ R_2_; bottom) (both on left); a more realistic schematic of the same computations based on actual cell types within the cortical microcircuitry (right). **(C)** Rall’s cable theory provided a biophysical model to understand how the passive properties of dendrites influenced synaptic integration (top left; C_m_, membrane capacitance; G_m_, membrane conductance; E_revsyn_, synaptic battery; G_syn_, synaptic conductance; r_i_, axial resistivity), and predicted strong location-dependent effect on synapses from different parts of the dendritic tree in models of neurons (bottom left). Subsequent models have been used to simulate how the location of a synapse influences how different inputs may be integrated at the soma (right panels); in this example, the somatic voltage change from an excitatory synaptic input to the distal apical or proximal basal dendrites is differentially affected by activation of a somatic (top simulations) or apical dendritic inhibitory synapse (percent reduction in somatic amplitude is shown on right for each simulation). Left panels in **(C)** are modified with permissions from Magee (2000) and right panel of **(C)** are modified with permission from Stuart et al. (2016).

Since the foundational insight of the neuron doctrine made by Cajal over 100 years ago (Cajal, 1906), we have known that the brain’s circuits are made up of many distinct cell types that are interconnected in intricate and complicated ways. The idea that the organizational topology of these cell types within a circuit can produce different logical computations was advanced in theoretical work by Warren McCulloch and Walter Pitts in the early 1940s (McCulloch and Pitts, 1990), who used simple yet elegant circuit motifs to formalize the relationship between circuit architecture and logical operations. At a finer scale, the work of Rall (1962, 1964, 1967), beginning in the 1960s, developed cable theory with the goal of understanding how the computations of individual neurons are governed by the biophysical properties of their branching dendrites and the spatial and temporal pattern of their synaptic inputs (see Figures 1A–C).

Much progress has been made in the subsequent decades to address the simplifications made in these pioneering studies in order to paint a clearer picture of how the properties of the brain emerge from its constituent parts. The overarching goal of neurocartography is to approach a mechanistic understanding of brain function from a structural perspective by developing maps of the nervous system in terms of cell types, their synaptic connections, and the location of molecules that permit synaptic communication and plasticity (Kasthuri and Lichtman, 2010). Just as Renaissance-era cartographic expeditions of the New World entailed some risk for an uncertain profit, the ultimate value of neurocartography for neuroscience is to date unclear but remains a promising direction to explore. A neurocartographic map of all connections in a mammalian brain (commonly referred to as a “connectome,” including chemical and electrical synapses) will be of maximal utility if it can differentiate among competing models of circuit architectures, as well as providing new, testable hypotheses of circuit functions once circuit diagrams are mapped and described. Given the non-linear nature of neuronal circuit computations, connectomes may fail to accurately predict function solely from structure in many cases; this issue is further complicated by the remarkable dynamics of the nervous system on both short and long timescales that would not be visualized by a map created from a single snapshot in time. Thus, even a complete neurocartographic map represents a lower bound of the computational capability of a neural system. Despite the inherent uncertainty and tremendous risks, Renaissance-era cartographic exploration of the new world dramatically changed the flow of goods and services and was ultimately fundamental in the creation of the global marketplace. Similarly, a high-resolution map of the structure of the nervous system may come to change how we view the mechanisms driving brain function.

Generating and analyzing a connectome is a remarkably tall order for any organism, and particularly challenging for mammalian brains given their complexity, size, and number of synaptic connections (Lichtman and Denk, 2011; Briggman and Bock, 2012). Even the mouse brain, which we focus on here and represents the smallest brain of the mammalian model organisms, contains an estimated 75 million neurons connected via one trillion synapses spread over 500 mm^3^. The ultimate goal of neurocartography, to generate a connectome of the human brain, will need to contend with an estimated 86 billion neurons (Herculano-Houzel, 2009) connected through 1,000 trillion synaptic connections. To generate a neurocartographic connectome, it’s necessary to be able to visualize and follow the pathways of the finest axonal “wires” through the dense neuropil. Since the finest axonal processes in the mammalian brain are below the resolution of traditional light microscopy (LM), electron microscopy (EM) has become the tool of choice for neurocartography in terms of mapping synapses. Although EM preparation protocols produce a distorted view of the nervous system (due to the inherent fixation and often dehydration of the tissue), arguably the largest disadvantages to EM-based approaches are the technical, financial, and temporal costs (Briggman and Bock, 2012). Given this challenge, a major focus of neurocartography has been the innovation and automation necessary to scale up the capability of EM to larger and more complete tissues as rapidly as possible. Such automation has already proven fruitful as shown by the publication of progressively larger EM volumes of the mammalian retina (Briggman et al., 2011; Helmstaedter et al., 2013; Ding et al., 2016), the entire larval brain of the fruit fly (Ohyama et al., 2015) and zebrafish (Hildebrand et al., 2017), and the complete adult fly brain (Zheng et al., 2018). Advances on all fronts have neurocartography poised to make rapid progress in the near future. These include whole brain *en bloc* sample preparation (Mikula et al., 2012; Mikula and Denk, 2015), creative new automated section collection schemes using “hot knife” (Hayworth et al., 2015) or tape collection methods (Kasthuri et al., 2015) that enable volumes to be collected with minimal tissue loss, computer-vision aided reconstruction algorithms (Kasthuri et al., 2015), and model building from connectomic datasets (Tschopp et al., 2018).

**FIGURE 2:**
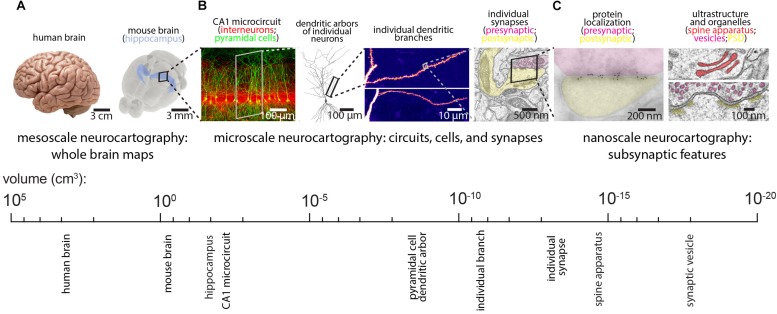
Spatial scales for mammalian neurocartography. **(A)** The ultimate goal of neurocartography is to create maps of connectivity across the entire brain. Given the immense volume and complexity of the human brain, most neurocartographic efforts have focused on surpassing technical hurdles and obtaining first principles from the mouse brain. We consider maps of long-range neuronal projection systems across the entire brain to be mesoscale neurocartography. **(B)** We consider maps of connectivity that span entire neural circuits to individual dendrites containing many synapses to be microscale neurocartography. **(C)** We consider maps that examine the localization of individual proteins or other molecules within synapses or measure the subsynaptic ultrastructural features to be nanoscale neurocartography. Panels are modified with permissions from Bloss et al. (2016, 2018) and unpublished work.

Despite this progress, the daunting task of reconstructing mammalian brains suggests that generating an EM-resolution mammalian connectome may not be feasible for decades. In its place, existing technologies can effectively complement and perhaps guide future EM-based neurocartography efforts, while emerging technologies can be tailored and applied to specific questions. For example, LM-based methods of synapse mapping can be validated by imaging the same sections with EM (see the Array Tomography (AT) section below for more discussion). In this way, smaller EM volumes can complement larger and more rapidly acquired LM volumes. Since the relevant spatial scale for neurocartography spans the mesoscale (e.g., long-range projection systems on the order of millimeters), the microscale (e.g., the dendritic arbors of individual neurons and their synaptic connections, on the order of tens to hundreds of micrometers), and the nanoscale (e.g., the precise features of the subsynaptic ultrastructure or localization of individual synaptic proteins, on the order of tens to hundreds of nanometers), a variety of techniques will facilitate neurocartography (see Figures 2A–C). Here, we review some of these LM based techniques and highlight the utility of these approaches for specific neurocartographic questions within the context of the mouse brain. Moreover, we discuss some of the recent data generated with these methods and suggest some important principles of circuit organization that can easily be tested with subsequent EM-based neurocartography.

## Light-Level Approaches for Mammalian Neurocartography

### Mesoscale Neurocartography

Is there a canonical circuit motif that is repeated across all cortical structures, or does connectivity within circuits vary depending on their precise cortical area? How does the subset of synaptic inputs received by a neuron relate to the specific pattern of its axonal projections? Where exactly do circuits processing one modality of information converge with circuits processing information from a different modality? Mesoscale-level neurocartography approaches permit such questions to be examined in a cell-type specific manner over the entire extent of the mammalian brain.

By necessity, mesoscale neurocartography approaches rarely visualize synaptic connectivity directly, but rather assay the potential for connectivity based on axonal projection patterns, axon bifurcations, and the presence of axonal *en passant* boutons. Although the supposition that axonal-dendritic overlap is sufficient to describe connectivity patterns (commonly referred to as Peter’s rule (Peters and Feldman, 1976)) frequently fails to predict the actual connectivity patterns between adjacent axons and dendrites (Mishchenko et al., 2010; Kasthuri et al., 2015), the spatial overlap of processes remains a necessary condition for a synaptic connection. As a result, mesoscale-level efforts are useful for identifying both local and long-range potential connectivity patterns within the brain (Hunnicutt et al., 2014; Oh et al., 2014).

Of the methods that are capable of examining neurons and their potential connectivity across the whole mouse brain, one-photon microscopy (e.g., widefield or confocal microscopy) is widely used because of its availability and versatility. One-photon imaging is compatible with a large number of tissue preparations and permits multiple fluorophores to be imaged. These features dovetail nicely with the expansive toolkit of genetically encoded fluorophores (Shaner et al., 2005) that can be expressed by neurons in a cell-type specific manner, enabling one-photon imaging methods to be combined with viral transsynaptic approaches such as anterograde tracing with stomatitis virus or retrograde monosynaptic tracing with modified rabies (Arenkiel and Ehlers, 2009; Luo et al., 2018), including variants that has been engineered to be more efficient and less cytotoxic (Reardon et al., 2016). For such volumes to span the entire brain, however, serial sections must be prepared, imaged, and aligned (e.g., as in (Hunnicutt et al., 2014)) owing to the light scattering properties inherent to thick slices or whole brain samples. In smaller volumes, on the order of individual cells, high-resolution confocal images can be used for correlative light and electron microscopic mapping of defined inputs (Schoonover et al., 2014) similar to more advanced preparations (e.g., AT; see discussion below).

**FIGURE 3:**
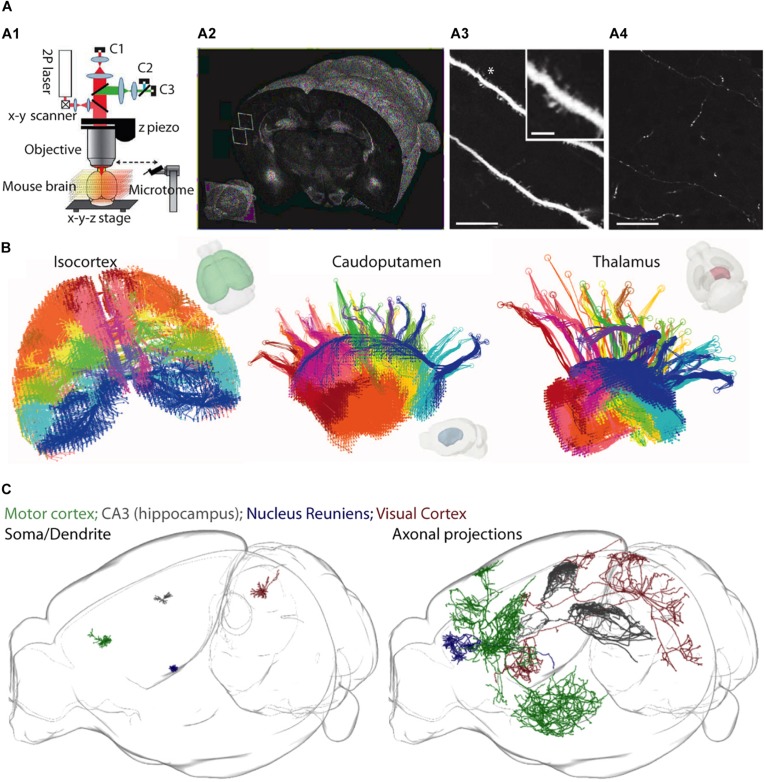
A two-photon serial tomography approach for mesoscale neurocartography. **(A1–A4)** The integration of a two-photon microscope with a vibratome **(A1)** permits serial imaging of fluorescent signals throughout the entire brain **(A2)** at resolution high enough to resolve dendrites **(A3)** and axons **(A4)**. Scale bars represent 25 μm in **(A3,A4)** and 5 μm (inset in **A3**). **(B)** A mesoscale projectome of distinct cortical regions (left) that make topographically organized projections to caudoputamen (center) and to regions of the thalamus (right). **(C)** Sparse labeling strategies permit the reconstruction of dendrites (left) and entire axonal projections (right) of individual neurons across the whole brain. Panel **(A)** modified with permissions from Ragan et al. (2012); panel **(B)** modified with permissions from Oh et al. (2014) and panel **(C)** created courtesy of MouseLight project at Janelia Research Campus: (http://ml-neuronbrowser.janelia.org/).

An exciting alternative approach has recently been developed that circumvents some of these issues. Two-photon serial tomography, in which mouse brains are imaged on a high-speed two-photon microscope equipped with a vibratome slicer, permits fluorescently labeled structures to be mapped across the entire brain at high resolution (Ragan et al., 2012) (see Figures 3A–C). In this approach, tiled two-photon images are acquired from a thick layer of tissue near the surface of a sample, and then the corresponding imaged volume is removed by the vibratome before a subsequent layer is imaged [analogous to serial block-face scanning EM (Denk and Horstmann, 2004)]. When this is performed sequentially, through the whole mouse brain, one can visualize the detailed path of populations or individual neurons and their corresponding axonal projections throughout the brain.

This is fundamentally different from the vast majority of tract-tracing experiments performed in the past, in which bulk injections led to a coarse description of the projection pathways in the brain. Already, this new approach has visualized populations of axon tracts of anatomically defined projection neurons (Oh et al., 2014), and been extended to individual axon projections of single neurons (Economo et al., 2016) (also see a resource of >1000 reconstructed individual neurons^1^). The major advantage is the ability to unambiguously map out the regional projection targets of individual neurons, permitting experiments aimed at untangling how information is routed out of one circuit and into another at the level of single neurons. At the same time, one drawback of two-photon serial tomography, as it is implemented now, is that it neither reveals synaptic connections nor identifies the postsynaptic neuron classes. However, this approach could be combined with higher resolution, microscale approaches (e.g., rabies tracing, AT, or EM) to map synaptic connections between defined cell types across the brain.

### Microscale Neurocartography

Neurocartographic efforts at the microscale are focused on understanding the fundamental relationship between the dendritic organization of synaptic inputs and the computations performed by a neuron (see Figure 2). Because synaptic inputs are first integrated locally in individual dendritic branches, where their voltage signals are shaped by a diverse set of ionic conductances, single dendritic branches act as individual integrative compartments of the neuron (Branco and Hausser, 2001; Poirazi and Mel, 2001). Delineating the underlying structural organization of excitatory and inhibitory inputs onto the dendrites is thus critical for gaining insight to the input-output transforms that cells can perform.

**FIGURE 4:**
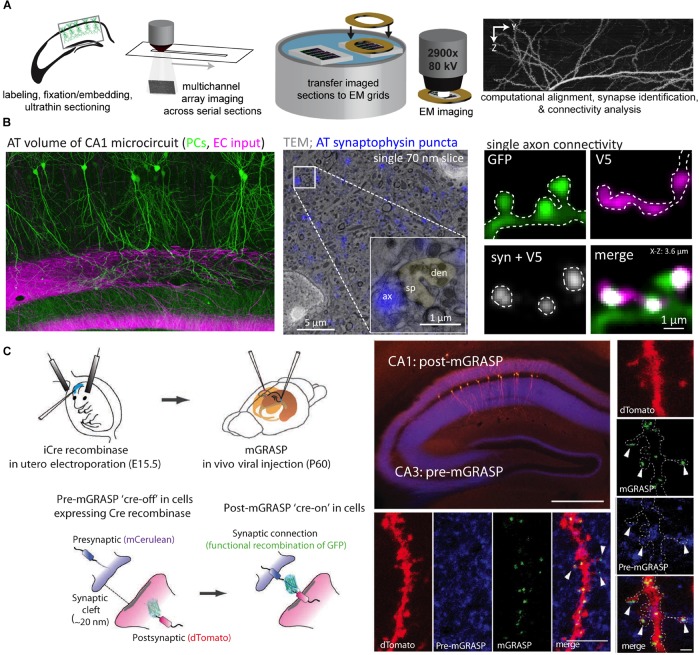
Array tomography and mGRASP permit the examination of synaptic connectivity at the microscale. **(A)** An array tomography pipeline that takes whole-brain samples with fluorescently labeled neuron populations and produces high-resolution yet large volumes of the underlying circuitry. Samples are made into planarized arrays, stained and imaged with a light microscope, and then sections are transferred to grids and imaged with an electron microscope. After imaging, volumes are computationally stitched together to produce volumes for connectivity analysis. **(B)** Example of an array tomography volume from area CA1 of the mouse hippocampus with pyramidal cells in green and excitatory inputs from the entorhinal cortex that target the distal dendrites in magenta (left); an example of correlative array tomography-electron microscopy sample with synaptophysin puncta in blue (inset shows a single asymmetric synapse with a dendritic spine pseudocolored green) (center); an example of a small array tomography stack with a single excitatory axon making a cluster of connections onto dendritic spines along a small portion of a postsynaptic dendrite (V5 refers to the epitope tag expressed in the afferent axon) (right). **(C)** mGRASP takes advantage of cell-type specific pre- and postsynaptic labeling strategies (*in utero* electroporation shown here) to visualize connectivity via the functional recombination of the GFP protein between pre- and postsynaptic populations (shown in cartoon in left panels). Examples of pre-mGRASP in hippocampal CA3 and post-mGRASP in area CA1 (top, scale bar is 500 μm), a branch segment from all three fluorescent channels (bottom four panels, scale bar is 1 μm), and a zoomed in example of clustered inputs (right vertical panel, scale bar is 1 μm). Panels **(A,B)** are modified with permissions from Bloss et al. (2016, 2018); Panel **(C)** modified with permissions from Kim et al. (2012).

One of the more versatile approaches for microscale neurocartography is AT, which was originally developed to probe the molecular phenotype of cortical synapses (Micheva and Smith, 2007) (see Figures 4A,B). In the initial demonstration, Micheva and Smith (2007) combined the basic elements of EM preparation (i.e., fixation, resin embedding, ultrathin tissue sectioning) with fluorescent imaging to examine up to 12 molecular targets in cortical tissue using a serial, multiplexed antibody labeling approach. Serial ribbons of ultrathin sections, which yield subdiffraction z-axis resolution, are collected as planarized arrays on coverslips and permit depth-independent labeling and imaging through large tissue volumes. By subsequently imaging the same ultrathin sections in an EM, this preparation permits fluorescently labeled structures to be directly placed in context of the local cortical ultrastructure.

The advantages associated with AT imaging include increased z-axis resolution, molecular multiplexing, sparse and selective labeling of defined neuron types, and the compatibility with correlative light-electron microscopic imaging. Moreover, the clustering of synaptic inputs (Rah et al., 2013; Bloss et al., 2018) and the measurements of the sizes of individual synapses can be reliably obtained (e.g., volumes of individual dendritic spines (Bloss et al., 2016)– though the smallest spines may still remain unresolved). These features permit the circuit identity of most synapses to be mapped along with some description of their molecular phenotype (Micheva et al., 2010) and a structural correlate of their strength (Matsuzaki et al., 2001). The selective labeling of defined neurons using epitope tags (Viswanathan et al., 2015) or fluorophores based on developmental timepoint (via *in utero* electroporation), brain region (via virus injections), or cell-type (through the use of Cre-expressing mouse lines and Cre-dependent viral constructs) circumvents a major issue associated with conventional EM-based neurocartography, where the majority of the neuronal processes (dendrites or axons) that coarse through a given volume cannot be assigned to a particular cell type [though see (Atasoy et al., 2014; Joesch et al., 2016) for new EM labeling techniques that address this issue]. Lastly, the ability to move between light and EM imaging modalities means that the accuracy of detecting synapses using fluorescent signals can be directly validated by EM (Collman et al., 2015).

This last point circumvents the primary disadvantage of AT for neurocartography (and light-level approaches in general), which is that synaptic connections are primarily inferred by the spatial colocalization of synaptic markers rather than defined by ultrastructure. This issue is compounded by the dependence of synaptic labeling on antibodies, which can be non-specific or only label a subset of synaptic structures. Correlative light-EM experiments, which should be performed for each antibody at each synapse type, have consistently demonstrated that AT can accurately identify bona fide synaptic connections with low false-positive rates (Micheva et al., 2010; Rah et al., 2013; Collman et al., 2015; Bloss et al., 2016).

An alternative light-level approach for the analysis of synaptic connectivity avoids the issue of spatially resolving synaptic connections but rather defines them based on the functional complementation between two split, non-fluorescent GFP fragments; this approach is called GFP reconstitution across synaptic partners (GRASP) (Feinberg et al., 2008). In the version optimized for mammalian synapses (mGRASP) (Kim et al., 2012), presynaptic neurons are infected with a virus encoding a portion of the GFP protein targeted to presynaptic sites and fused to a fluorescent protein in order to visualize the axonal cytoplasm. Postsynaptic neurons are engineered to express the complementary split portion of GFP trafficked to postsynaptic sites along with a second fluorescent cytoplasmic protein. The functional fluorescent GFP protein that is produced when the two split constructs bind at putative synaptic sites permits the visualization of synaptic connections (See Figure 4C). Recent mGRASP variants have expanded on this original configuration (called eGRASP or dual eGRASP) by modifying the GFP protein in order to alter its emission spectrum, permitting two sets of synapses to be mapped at once (Choi et al., 2018).

The advantage of mGRASP is that synapses can be mapped across a large portion of a microcircuit including many neurons (Druckmann et al., 2014). Since the pre- and postsynaptic mGRASP components can be selectively targeted to cell types as in AT (Kim et al., 2012), this technique enables high throughput mapping of defined sets of pre-and postsynaptic populations (Druckmann et al., 2014). Also, like AT, axons of a given cell type or projection class with cell bodies outside the imaged volume can be easily identified based on fluorescent reporter expression. Unlike AT, however, putative mGRASP synaptic signals have not been directly verified by correlative EM, are less amenable to multiplexed analyses of the molecular synaptic phenotype, and are dependent on overexpression of mutated synaptic proteins that may change synaptic dynamics (i.e., may favor synapse formation or synapse turnover). Furthermore, mGRASP may require modifications to achieve synapse-specific targeting and GFP recombination to match the different dimensions of the synaptic cleft across disparate synapse types.

### Nanoscale Light-Level Neurocartography

**FIGURE 5:**
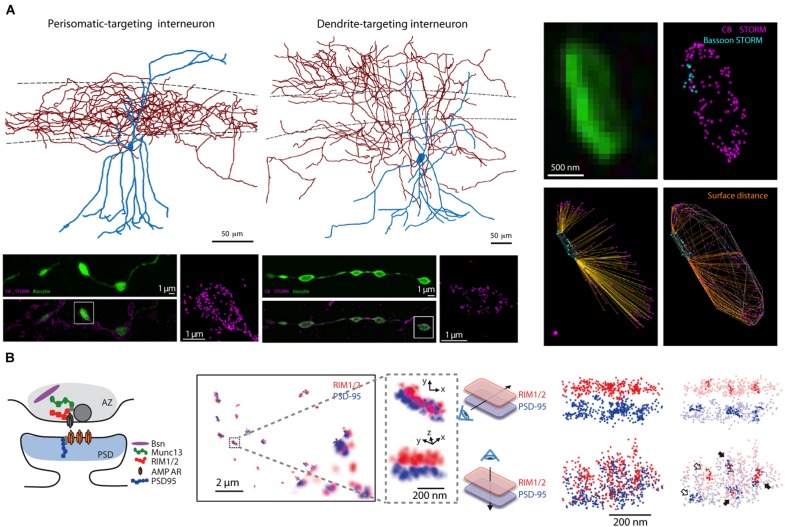
Super-resolution light microscopy imaging yields nanoscale maps of proteins within individual synapses. **(A)** Two types of interneurons (top left), targeting the soma or dendrites of CA1 pyramidal cells, express cannabinoid type-1 receptors in their presynaptic terminals as shown by STORM imaging of filled boutons (bottom left panels, vertically aligned to cell type); the precise location of CB1 proteins within individual boutons can be mapped in relation to the active zone location (assessed by STORM imaging of the active zone protein bassoon, right panels). **(B)** STORM imaging reveals a putative nanocolumnar modular organization of synaptic proteins within individual synapses. A schematic cartoon of the putative location of synaptic proteins at the synapse (left), RIM1/2 and PSD-95 proteins imaged by STORM at synapses between cultured neurons (bottom corner shows corresponding widefield images of these two proteins, inset is magnified to the right), and co-clusters of RIM1/2 and PSD-95 identified by the high density of STORM localization within pre- and postsynaptic sites (right). Panel **(A)** is modified with permissions from Dudok et al. (2015) and panel **(B)** is modified with permissions from Tang et al. (2016).

Is there a spatial organization of spine size and associated strength of synapses along neuronal dendrites? Do synaptic strengths vary as a function of the afferent cell type providing the input? Do molecules that are important for synaptic function, such as neurotransmitter receptors and plasticity-related proteins, show equal expression at all synapses? These fundamental neurocartographic questions lie at the nanoscale, which for the last few decades has been almost exclusively investigated with traditional EM approaches (Harris and Weinberg, 2012) or with postembedding immunoelectron microscopy (Amiry-Moghaddam and Ottersen, 2013). Relatively new forms of super-resolution LM such as structured illumination microscopy (Gustafsson, 2005), stimulated emission depletion (STED) microscopy (Klar and Hell, 1999), super-resolution shadow imaging (Tonnesen et al., 2018) (also known as SUSHI) and photoactivated localization microscopy (PALM) (Betzig et al., 2006) [also called stochastic optical reconstruction microscopy (Rust et al., 2006) or STORM] (see Figures 5A,B) have been developed that surpass diffraction-limited resolution. These can potentially be useful for nanoscale neurocartographic studies aimed at understanding rules that govern the molecular landscape or temporal dynamics of individual synapses. However, since they are often limited to the superficial aspects of a slice, section, or cultured monolayer, super-resolution techniques generally have not been used for larger scale connectivity studies *ex vivo*.

An emerging technology capable of simultaneously resolving cell-type specific connectivity and the nanoscale organization of synaptic proteins is expansion microscopy (Chen et al., 2015). Expansion microscopy physically enlarges samples through delipidation, protein digestion, and infiltration of a swellable polymer (e.g., sodium polyacrylate). When the sample is subsequently transferred to an aqueous solution, the gel expands several-fold in an isotropic manner. Expanded samples permit diffraction-limited imaging to resolve structures that remain poorly resolved in naïve samples. Although some fraction of tissue protein is digested during the protocol, expanded samples remain compatible with antibody staining, which permits synaptic markers and reporter fluorophores to be visualized with antibody amplification. Efforts to utilize expanded samples for connectivity work have only just begun (Gao et al., 2018); however, the incompatibility with correlative EM imaging that might validate connectivity results from expansion microscopy remains a substantial hurdle that will need to be addressed in the future.

Although these light-level super-resolution technologies are powerful nanoscale approaches in their own right, they are still maturing and their optimal application to neurocartography is not yet entirely clear. In some cases, experiments can utilize separate but related datasets to place EM results within the context of ultrastructure (i.e., see (Bloss et al., 2018) for the combination of AT and EM to resolve the circuit identity of specific connections found), but methods that permits meso- and microscale data to be combined with that at the nanoscale level from a single sample remains a significant challenge and a major goal for the future.

### Live Neurocartography: Functional Mapping of Synaptic Connections

The above approaches all seek to provide an anatomical framework to constrain the repertoire of possible computations supported by circuit motifs; although such data is invaluable, functional measures of synaptic connectivity are also necessary to examine how the synaptic elements of the circuit might influence cellular integration. Moreover, determining the functional properties or relative strengths of specific synapses, including the release probability and short-term plasticity, are critical factors that influence cellular computations. For these measures the most appropriate approach is electrophysiology, where these features can be assayed directly by patch clamp recordings in acute brain slices.

Classical methods involving paired intracellular recordings in brain slices underestimate connectivity because of severed long-range connections. Furthermore, the use of stimulating electrodes placed near an afferent pathway to electrically stimulate en masse while recording intracellularly from a postsynaptic cell lacks input specificity. To overcome these limitations optogenetic approaches like channelrhodopsin-2 assisted circuit mapping (CRACM) (Petreanu et al., 2007, 2009) can be used to functionally map the subcellular location of defined presynaptic inputs onto defined postsynaptic cell types. CRACM experiments typically involve restricting expression of the depolarizing channelrhodopsin-2 (ChR2) channel to a defined set of projection neurons using *in utero* electroporation, reporter transgenes, or via viral infection. Acute brain slices are made, and ChR2-expressing axons are excited with one-or two-photon light, while simultaneously making patch-clamp recordings from a nearby postsynaptic neuron.

The major advantage of CRACM is that it permits a fundamentally different type of synaptic map to be made between defined sets of presynaptic neurons and a defined postsynaptic neuron (typically identified by somatic location, physiological properties, and *post hoc* morphological reconstruction). In addition, the use of optical stimulation (rather than electrical) enables the experimenter to distinguish between monosynaptic and polysynaptic inputs by bathing slices in a cocktail of sodium and potassium channel blockers that suppress endogenous action potential electrogenesis, meaning only ChR2-expressing axons can drive responses in the recorded neuron. Unlike AT or mGRASP, results from CRACM experiments can determine a number of important functional features. For example, CRACM maps can determine whether the synapses activated within a defined presynaptic class have high or low release probabilities, or if they contain a specific set of receptor subtypes compared to other synapses. However, the lack of a postsynaptic response to an optical stimulation cannot differentiate between a silent synapse or a lack of synaptic input. Combining techniques of activating and recording from neuronal subsets purely using optogenetic sensors and activators has given rise to all-optical electrophysiological approaches, which potentially can map connectivity across large numbers of defined sets of neurons *in vitro* or *in vivo* (Emiliani et al., 2015; Packer et al., 2015).

Compared to the anatomical maps produced by AT or mGRASP approaches, what does a CRACM-based map look like? On one hand, synaptic connections that have been presumed to be specific to a cell type can be mapped using full-field illumination. For example, CA3 pyramidal cells are defined in part by the receipt of strong, facilitating synaptic input from mossy fibers. The recent finding by CRACM experiments that there is an additional subset of pyramidal cells that lack such input demonstrates the utility of this technique to map functional synaptic connections in an all-or-none manner (see Hunt et al., 2018). On the other hand, CRACM can be used to map more graded patterns of input onto localized portions of the dendrite. In this case, precise input locations are marked by the location where the photostimulation produced a postsynaptic response (Petreanu et al., 2009). In this type of experiment (and in contrast to the binary maps produced by full field illumination in Hunt et al. (2018), the resolution of a CRACM map is limited by the photostimulation pattern which is typically spot sizes on the order of tens of micrometers (Chiu et al., 2013). By using photostimulation around dendritic segments that lack intervening branch points, where somatic responses during stimulation will reflect inputs to that segment only, the spatial resolution has been estimated to be approximately 60 μm (Petreanu et al., 2009). To some extent the resolution of a map can be improved by utilizing two-photon calcium imaging of photostimulation-induced activity in dendritic spines (Little and Carter, 2012). However, using CRACM to compare the strength of different input by comparing the amplitude of the postsynaptic current is problematic because of the inherent problems of using somatic voltage-clamp to interpret electrotonically distant synaptic events (Williams and Mitchell, 2008; Beaulieu-Laroche and Harnett, 2018). Despite these caveats, CRACM-style experiments still provide the best means to produce functional neurocartographic maps at subcellular resolution.

## What Principles Have We Learned From These Approaches?

A mouse brain connectome would permit new insights to the specificity of synaptic connections between defined cell types and to whether higher-order, structured input patterns are embedded within the overall circuitry. Can such information actually provide data regarding the functional properties of a circuit? A recent example hinting at this possible outcome can be found in a portion of an invertebrate EM-based connectome, which has provided evidence that computations of a circuit can be inferred from a detailed synaptic wiring diagram (Tschopp et al., 2018). In the absence of such a connectome for the mouse, what have we learned from these alternative neurocartographic approaches regarding the cell-type specific wiring of the brain’s circuits? Is there evidence for structured forms of connectivity between cell types, and if so, at what spatial scales: at the cellular level, at the level of individual dendritic branches, or at the sub-branch level? Lastly, what results from existing neurocartographic studies would permit us to ask more pointed questions once an EM connectome is in hand?

### Mesoscale Neurocartography Suggests a Variety of Circuit Topologies

The versatility of mesoscale approaches (e.g., anterograde and retrograde tracing, sparse and multicolor cell-type specific labeling strategies) has revealed a remarkable heterogeneity in how connectivity in the brain is organized. Recent results from a two-photon serial tomography approach using viral GFP expression to map the neocortical mesoscale projectome (Oh et al., 2014) have produced a landmark analysis of cortical circuit organization. This work, using quantitative modeling approaches to dissect a large and comprehensive anatomical dataset, demonstrates the existence of parallel pathways that mediate the routing of information to their spatially distinct regions of the basal ganglia and thalamus (shown in Figure 3B). Similarly, anatomical results from the hippocampus have demonstrated a largely parallel organization governing the flow of information through hippocampal CA1 microcircuitry. Pyramidal neurons located in the proximal portion of CA1 receive input from the medial entorhinal cortex and project to the distal subiculum, while pyramidal neurons located in the distal part of CA1 receive input from the lateral entorhinal cortex and project to the proximal subiculum. These subcircuits appear genetically organized (Berns et al., 2018) and process different streams of information (Knierim et al., 2006).

Results from mesoscale, transsynaptic rabies mapping have suggested that cortical projections neurons have distinct sets of inputs, which differs from the broad input organization of the noradrenergic neuromodulatory system (Schwarz et al., 2015) [though see (Kebschull et al., 2016) for a different result]. In another notable study, Betley et al. (2013) used a retrograde viral strategy to demonstrate that a molecularly defined class of hypothalamic neurons use a parallel, one-to-one projection strategy to communicate with individual downstream targets. Thus, mesoscale efforts to map the organization of projection systems have demonstrated that the brain uses a variety of wiring strategies to transmit information. Future work at the mesoscale level may ultimately aid in the interpretation of magnetic resonance imaging (Toga et al., 2006) and electroencephalography results (Petersen and Sporns, 2015) that aim to unite how the anatomical features of the brain support cognition. A powerful approach to extend such data could be the combination of mesoscale technologies (e.g. two-photon serial tomography) with those that can determine the postsynaptic target identities at the microscale or with subsynaptic precision at the nanoscale. Even in the absence of that combination, mesoscale circuit-mapping strategies should continue to provide interesting hypotheses that can be confirmed and extended once a synaptic-scale EM connectome is in hand.

### Microscale Efforts Reveal Different Forms of Cell-Type Specific Synapse Targeting

Neurocartographic efforts at the microscale are aimed at understanding whether structured (i.e., non-random) wiring patterns are employed by distinct circuit elements and support specific cellular computations. This question has been addressed in the CA1 area of the hippocampus, in large part because of the interest in gaining mechanistic insight toward the mnemonic functions of the hippocampal formation. Druckmann et al. (2014) used mGRASP to examine connectivity between broadly-labeled presynaptic CA3 populations and CA1 neurons that are sparsely labeled with the postsynaptic mGRASP construct. If connectivity were random, then neighboring CA1 neurons should have comparable numbers of mGRASP^+^ inputs; however, the authors found an unexpectedly large difference in the mGRASP+ excitatory input number on the apical and basal branches, arguing against random CA3-to-CA1 connectivity. The synapse numbers on these branches frequently differed from the predicted number of synapses based on a Poisson model, and inputs on single branches were found to cluster at a higher rate than predicted (i.e., large numbers of synapses had intersynapse distances less than 1.5 μm). These results suggest wiring mechanisms governing connectivity within the CA3-to-CA1 pyramidal cell pathway produce both branch-level and subbranch non-uniformities. Interestingly, such synaptic structure is not present in the same CA3 projection pathway onto CA1 interneurons. A recent study (Kwon et al., 2018) using the same broad mGRASP labeling of CA3 presynaptic partners, but with postsynaptic mGRASP expressed only in CA1 PV-Cre interneurons, found very few postsynaptic branches containing excitatory synapses that deviated from that predicted by a Poisson model. Thus, structured connectivity appears to be selective within efferent systems targeting pyramidal cells.

An altogether different form of structured dendritic connectivity was found by using AT to examine inhibitory synaptic connections onto pyramidal cells within area CA1 (Bloss et al., 2016). When the connectivity of molecularly defined sets of local CA1 interneurons was measured onto the dendrites of CA1 pyramidal cells, dendritic branches of the same order received a similar density of input from each class of defined GABAergic interneurons, but large heterogeneities in synaptic connectivity were evident across different branch types. This suggests a precise form of cell-type specific dendritic targeting. In addition, the synaptic innervation pattern of some GABAergic cell types exhibited strong subbranch targeting, forming synaptic connections preferentially near the branch point origin or at the branch point end. Simulations of these anatomical results in morphologically detailed models of neurons demonstrated that these connectivity patterns alone enable differential control over the integration of excitatory input.

Do such structured connectivity patterns at the branch and subbranch levels generalize to other microcircuits in the brain? There is some limited evidence to suggest that they do; Rah et al. (2013) used AT to map the axons of thalamic neurons onto the dendrites of layer V neocortical pyramidal neurons and revealed clustered synapses along the basal dendrites. Future experiments should seek to extend results from the hippocampus to additional cortical and subcortical circuits. In any case, these initial neurocartography results demonstrating synaptic clustering strongly suggest several fruitful directions for quantitative investigation once a connectome is in hand.

### Nanoscale LM Efforts Uncover Synapse-Specific Anatomical and Molecular Rules

Neurocartography at the nanoscale has been dominated by studies using EM because of its inherent higher resolution (Harris and Weinberg, 2012) and ability to quantify proteins within the synapse (Amiry-Moghaddam and Ottersen, 2013). The super-resolution light-level techniques mentioned above have recently demonstrated their capability to answer some select neurocartography questions. Advantages of light-level nanoscale neurocartography include the potential for live slice preparations that can measure structural plasticity at identified synapses, and a larger repertoire with greater flexibility for multiple epitopes/fluorophores compared to multiple epitope EM immunogold labeling strategies. As examples, STED imaging has been used to show how the geometry of the neck of dendritic spines, which influences voltage responses at the synapse and in the dendrite, may be altered during epochs of synaptic plasticity (Tonnesen et al., 2014); and STORM imaging has demonstrated quantitative differences in the spatial organization of cannabinoid receptors at defined inhibitory connections (Dudok et al., 2015). Recent work has also shown that at excitatory connections, there is a putative “nanocolumn” organization of pre- and postsynaptic elements that are shaped coordinately by plasticity (Tang et al., 2016; Hruska et al., 2018).

Much less LM nanoscale work has examined these features within the context of identified circuit connections (i.e., from cell type “A” in brain region 1 onto cell type “B” in brain region 2). One approach toward this goal may be to obtain nanoscale data from EM experiments, then utilize light-level neurocartographic approaches in order to interpret the EM results within the context of previously identified neural circuitry. For example, several recent reports have found (using EM) that single axons can form multiple, “compound” synapses onto a target dendritic segment (Bartol et al., 2015; Kasthuri et al., 2015; Schmidt et al., 2017; Bloss et al., 2018). Such connections are interesting because they would produce spatiotemporal input correlations owing to their close proximity on the postsynaptic dendrite and shared presynaptic action potential patterns. Intriguingly, synapses that are part of a compound connection exhibit similar synaptic morphologies and subsynaptic attributes (e.g., pre- and postsynaptic organelles) (Bartol et al., 2015; Kasthuri et al., 2015; Bloss et al., 2018). However, these EM results have left the identity of the circuit or circuits forming compound synapses unknown. Using cell-type specific labeling of afferent projections via AT imaging it was possible to demonstrate that compound synapses in the hippocampal circuit arise from cortico-hippocampal but rarely in thalamo-hippocampal or intrahippocampal projections (Bloss et al., 2018). This combination of techniques suggests nanoscale neurocartography will not remain effectively dominated by EM. Rather, super-resolution technologies may provide a useful alternative to address nanoscale organizational principles simultaneously, especially when they can be combined with strategies that map molecular patterns and synaptic connections.

## The Bright Future of Neurocartography

An understanding of the structure-function relationship within the mammalian brain has remained elusive. This stems in large part because of the difficulties inherent in capturing the brain’s fine subsynaptic structures over volumes large enough to visualize whole brains, entire circuits, or even complete neurons. Significant advances in neurocartography have made remarkable progress, evidenced by the recent publication of the complete adult fruit fly brain imaged at synaptic resolution (Zheng et al., 2018), a milestone achievement that promises to transform the study of this model organism. Cajal could only have imagined being able to see neuronal structures in the detail now possible. Similarly, MacCulloch, Pitts, and Rall would all appreciate the progress made toward understanding how microcircuit elements are wired together to support computations performed by individual cells and circuits.

Electron microscopy reconstructions of tissue volumes from mammalian brains have gotten progressively larger yet remain far from the capability needed to produce a mesoscale map of the brain at nanoscale resolution. Two important reasons suggest this gap should only increase our excitement for the future of neurocartography. First, new light-level approaches that fill this gap continue to provide strong evidence that deconstruction of circuit connectivity is likely to be a fruitful avenue of research. Second, the pursuit of neurocartography continues to drive the development of creative new imaging technology and graph theoretical analyses that will enable the generalization of neurocartographic features across complex biological and artificial neural networks. As Sydney Brenner once said, “Progress in science depends on new techniques, new discoveries, and new ideas, probably in that order (Robertson, 1980),” the efforts needed for the advance of neurocartography may well prove him right.

## Author Contributions

EB and DH wrote the manuscript.

## Conflict of Interest Statement

The authors declare that the research was conducted in the absence of any commercial or financial relationships that could be construed as a potential conflict of interest.

## Footnotes

1 http://ml-neuronbrowser.janelia.org/
